# Multiomics Integration of Parkinson’s Disease Datasets Reveals Unexpected Roles of IRE1 in Its Pathology

**DOI:** 10.3390/ijms26146711

**Published:** 2025-07-12

**Authors:** Bianka Alexandra Pasat, Matthieu Moncan, Eleftherios Pilalis, Afshin Samali, Aristotelis Chatziioannou, Adrienne M. Gorman

**Affiliations:** 1 Apoptosis Research Centre, University of Galway, H91W2TY Galway, Ireland; 2 School of Biological and Chemical Sciences, University of Galway, H91W2TY Galway, Ireland; 3 Research Ireland Centre for Research Training in Genomics Data Science, University of Galway, H91W2TY Galway, Ireland; 4 Center of Systems Biology, Biomedical Research Foundation of the Academy of Athens, 4 Soranou Ephessiou Str, 11527 Athens, Greece; 5 e-NIOS Applications PC, 196 Al Syggrou Ave., 17671 Kallithea, Greece; 6 CÚRAM Research Centre for Medical Devices, University of Galway, H91W2TY Galway, Ireland

**Keywords:** Inositol-Requiring Enzyme 1 (IRE1), isoform usage, multiomics, Parkinson’s disease (PD), regulated IRE1-dependent decay (RIDD)

## Abstract

Parkinson’s disease (PD) is the second most common neurodegenerative disease. It primarily affects the motor system but is also associated with a range of cognitive impairments that can manifest early in disease progression, indicating its multifaceted nature. In this paper, we performed a meta-analysis of transcriptomics and proteomics data using MultiOmicsIntegrator to gain insights into the post-transcriptional modifications and deregulated pathways associated with this disease. Our results reveal differential isoform usage between control and PD patient brain samples that result in enriched alternative splicing events, including an extended UTR length, domain loss, and the upregulation of non-coding isoforms. We found that Inositol-Requiring Enzyme 1 (IRE1) is active in PD samples and examined the role of its downstream signaling through X-box binding mRNA 1 (*XBP1*) and regulated IRE1-dependent decay (RIDD). We identified several RIDD candidates and showed that the enriched alternative splicing events observed are associated with RIDD. Moreover, in vitro mRNA cleavage assays demonstrated that *OSBPL3*, *C16orf74*, and *SLC6A1* mRNAs are targets of IRE1 RNAse activity. Finally, a pathway enrichment analysis of both XBP1s and RIDD targets in the PD samples uncovered associations with processes such as immune response, oxidative stress, signal transduction, and cell–cell communication that have previously been linked to PD. These findings highlight a potential regulatory role of IRE in PD.

## 1. Introduction

Parkinson’s disease (PD) is a complex neurodegenerative disorder characterized by the progressive loss of dopaminergic neurons in the substantia nigra, leading to motor and cognitive impairments [1] and the presence of Lewy bodies [2]. The pathogenesis of PD involves multiple dysregulated pathways, including a loss of proteostasis, mitochondrial dysfunction, alterations in lipid metabolism, neuroinflammation, and altered cellular signaling mechanisms [3,4].

Previous findings have indicated a role of endoplasmic reticulum (ER) stress and activation of the unfolded protein response (UPR) in PD. In PD brain tissue samples, activation of the protein kinase (RNA)-like endoplasmic reticulum kinase (PERK) arm of the UPR has been reported [5]. Treatment of neuronal PC12 cells with the PD-mimetic drugs 6-hydroxydopamine (6-OHDA), 1-methyl-4-phenylpyridinium (MPP^+^), or rotenone activates the UPR, as seen through the phosphorylation of PERK and another ER stress sensor, Inositol-Requiring Enzyme 1 (IRE1), as well as induction of the ER chaperone Binding immunoglobulin protein (BiP) [6]. In MN9D cells, although genes involved in the UPR were induced by both 6-OHDA and MPP^+^, only 6-OHDA produced the spliced form of X-box binding mRNA (*XBP1*), indicative of IRE1 activation. [7].

Among the UPR signaling pathways, IRE1 has emerged as a key player in mediating cellular responses to ER stress. IRE1 is an ER transmembrane protein that functions as an ER stress sensor, activating downstream signaling pathways that can either promote cell survival or trigger apoptosis depending on the severity and duration of the stress [8]. The cytosolic part of the protein has both a kinase and an endoribonuclease (RNase) domain [9]. The RNase activity of IRE1 cleaves *XBP1* mRNA at two consensus sites, leading to the generation of spliced *XBP1* (XBP1s) mRNA [10,11], which is translated into a transcription factor that activates chaperones and proteins involved in protein folding and degradation, ultimately supporting cell survival [8]. Concurrently, IRE1 can also cleave other RNAs that have an XBP1-like motif via a process called regulated IRE1-dependent decay (RIDD), leading to their exonuclease-mediated degradation [10] and thus reducing the protein load in the ER. However, if stress persists, RIDD activity can also contribute to apoptosis [8]. This dual role of IRE1 allows cells to adapt to varying levels of ER stress through a complex, finely tuned interplay between its two signaling pathways.

We recently developed a pipeline, MultiOmicsIntegrator (MOI), for the analysis of single and multiple omics, as well as the integration of multiple-omics datasets to reveal pathways that are biologically relevant to a particular treatment or condition [12]. Here, we have used MultiOmicsIntegrator to perform an in-depth analysis of publicly available transcriptomic and proteomic datasets from PD patient samples [13]. Since the datasets were from a common source, we also sought to apply integration of the two omics layers in order to uncover the role of IRE1 in the pathogenesis of this disease.

## 2. Results

Our analysis was based on a previously published study [13] that included both transcriptomics and proteomics data. We selected samples with data available for both omics layers (12 control and 12 PD patient brain samples).

### 2.1. The Integrated Analysis Reveals Specific Changes to the Transcriptional Landscape in PD Patient Samples

**Figure 1:**
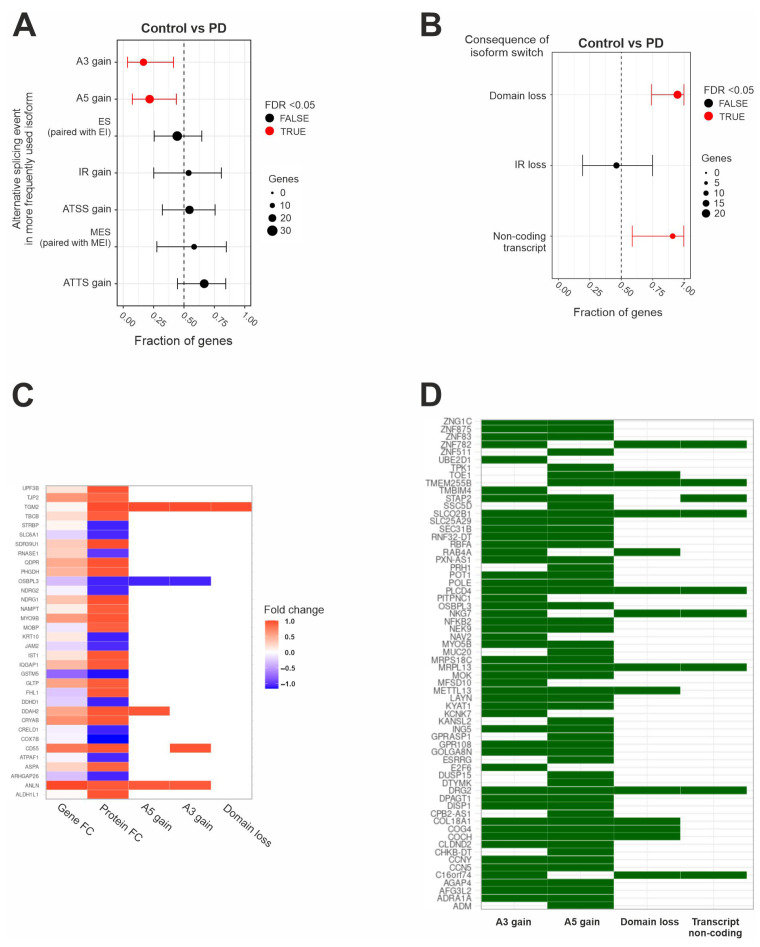
Transcriptional changes in isoforms in PD samples. (**A**) Enriched isoform switching events (increased 3′ UTRs and 5′ UTRs) occurring due to differential isoform usage. A3 gain, A5 gain, exon skipping (ES), intron retention (IR) gain, multiple exon skipping (MES), alternative transcript start site (ATSS) gain, and alternative transcript termination site (ATTS) gain are paired with A3 loss, A5 loss, exon inclusion (EI), MEI loss, IR loss, ATSS loss, and ATTS loss, respectively. The *X* axis displays the proportion of genes that have isoforms that display these events. (**B**) The consequences of differential isoform usage, as defined by the upregulation of transcripts that exhibit domain loss (paired with domain gain), IR loss (paired with IR gain), or non-coding transcript (paired with coding transcripts). (**A**,**B**) The *Y* axis is the fraction of genes with switches primarily resulting in the splicing event indicated, with 95% confidence intervals. (**C**) Common deregulated features across transcriptomics and proteomics with fold changes and enriched events from the isoform analysis in (**A**). (**D**) Integrated features retrieved using the MCIA and their distribution across enriched events from the isoform analysis in (**A**).

**Figure 2:**
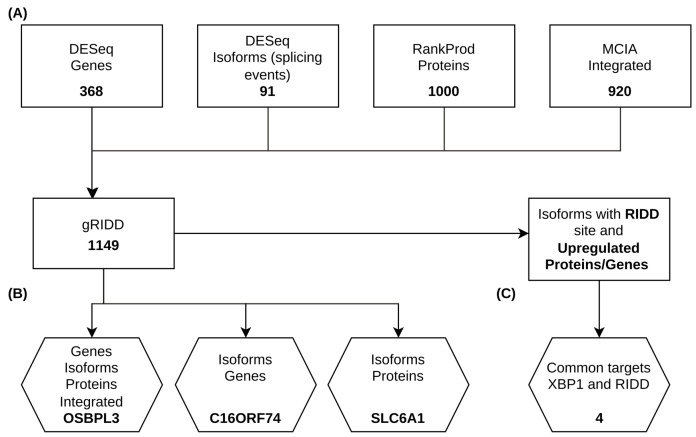
A schematic summarizing the outcomes at each analytical stage. Deregulated genes were defined using an FDR threshold < 0.05. Isoforms were selected based on the differential isoform fraction (dIF > 0.10), an FDR < 0.05, and the presence of at least one enriched splicing event. Proteins were considered deregulated if their *p*-value was <0.05 (**A**). RIDD and XBP1 targets were predicted with gRIDD (probability > 0.9) and FIMO (*p*-value < 0.0005), respectively. Features selected for experimental validation were prioritized based on their presence across multiple omics layers (**B**). Some features were predicted to be targets of both RIDD and XBP1 (**C**).

First, we examined the transcriptional landscape of patients with PD compared to that for control groups. Our focus was on gene isoforms, specifically investigating differential isoform usage and alternative splicing events. Figure 1A illustrates the fraction of genes displaying the splicing events caused by isoform switching. Among the observed splicing events, 3′ untranslated regions (A3) and 5′ untranslated regions (A5) were enriched. A gain in 3′ UTRs was observed in 13% of the genes, and a gain in 5′ UTRs was observed in 24% of the genes. Additionally, we analyzed the functional consequences of these isoform switches. Figure 1B shows that over 75% of the affected genes exhibited either upregulation of isoforms that lead to “domain loss” or upregulation of non-coding isoforms of these genes. Next, we assessed whether these post-transcriptional modifications were reflected in the proteomics data. The common significantly (with a *p*-value < 0.05) deregulated features across omics and their overlap with the enriched splicing events identified in the isoform analysis are shown in Figure 1C. Figure S1 depicts a Venn diagram of significantly (*p*-value < 0.05) deregulated features originating from the proteomics, transcriptomics, and isoform analyses. Significantly deregulated features include altered gene expression, altered protein expression, an increased length of A5, an increased length of A3, and domain loss (Figure 1C). Several of the genes exhibited changes in these features, i.e., Tight Junction Protein 2 (*TJP2*), Oxysterol Binding Protein-Like 3 (*OSBPL3*), DDAH Family Member 2, ADMA-Independent (*DDAH2*), the CD55 molecule (Cromer Blood Group) (CD55), and Anillin, an actin-binding protein (*ANLN*). Notably, *ANLN*, *DDAH2*, and *CD55* have previously been linked to PD [14,15,16], while *TJP2* and *OSBPL3* have roles in immune regulation and response [17,18]. Although identifying common features across omics datasets is a robust approach, it may overlook complex interaction patterns. To address this, we also applied a Multiple Co-Inertia Analysis (MCIA) and examined whether any of these features were associated with the splicing events described earlier (Figure 1D). Figure S2 provides a schematic overview of the analytical workflow underlying all of the results presented in this paper. Figure 2 depicts the features obtained at each analytical step. Among the two approaches, OSBPL3 emerged as the sole feature shared across both analyses.

### 2.2. IRE1 Has Higher Activity in Patients with PD

**Figure 3:**
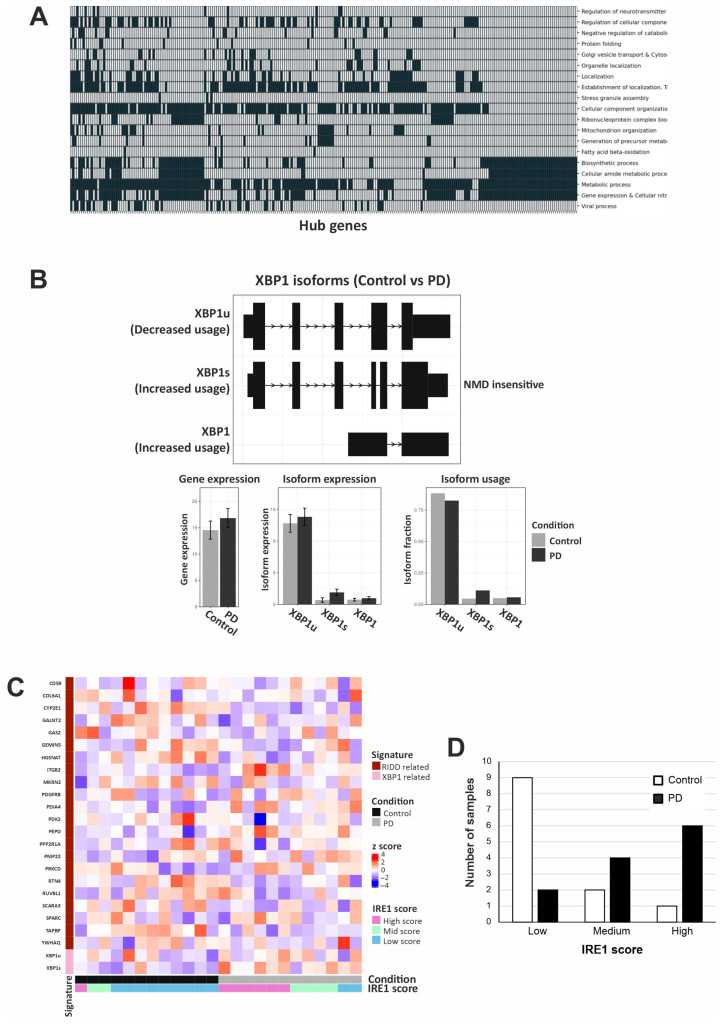
IRE1 activity is elevated in PD samples. (**A**) Heatmap of enriched biological processes of features integrated with MCIA. (**B**) *XBP1u* and *XBP1s* transcripts in control and PD samples. (**C**) Heatmap of IRE1 RIDD targets and *XBP1s/u* versus the relative IRE1 scores across patients and control groups. (**D**) Distribution of IRE1 calculated score across samples. The chi-squared analysis of the IRE1 scores and the condition of the samples showed that they were correlated, with a *p*-value = 0.013.

Protein folding is a tightly regulated biological process that plays a key role in maintaining proteostasis, and its dysregulation is implicated in various neurodegenerative diseases [19]. The pathway enrichment analysis of the MCIA-integrated features revealed that protein folding is amongst the deregulated processes in PD (Figure 3A). We also found that patients with PD exhibit differential isoform usage of the *XBP1* gene (Figure 3B). Specifically, the usage of the transcript corresponding to *XBP1s* was elevated, whereas the usage of the transcript corresponding to unspliced XBP1 (*XBP1u*) was decreased in PD, patterns that are in accordance with elevated IRE1 activity (Figure 3B). To explore IRE1 activity further, we analyzed the expression of previously reported RIDD targets. Then, we used the relative expression of these RIDD targets together with the expression of the *XBP1* transcripts and calculated the IRE1 activity scores for PD patient and control samples (Figure 3C). We then performed a chi-squared analysis, which revealed a statistically significant association between the IRE1 activity categories (high, medium, low) and sample classification (control vs. PD) (Figure 3D). While this analysis does not establish causality, the observed association is consistent with increased IRE1 activity in the PD cohort compared to that in controls.

### 2.3. IRE1’s RIDD Activity and Differential Isoform Switching

**Figure 4:**
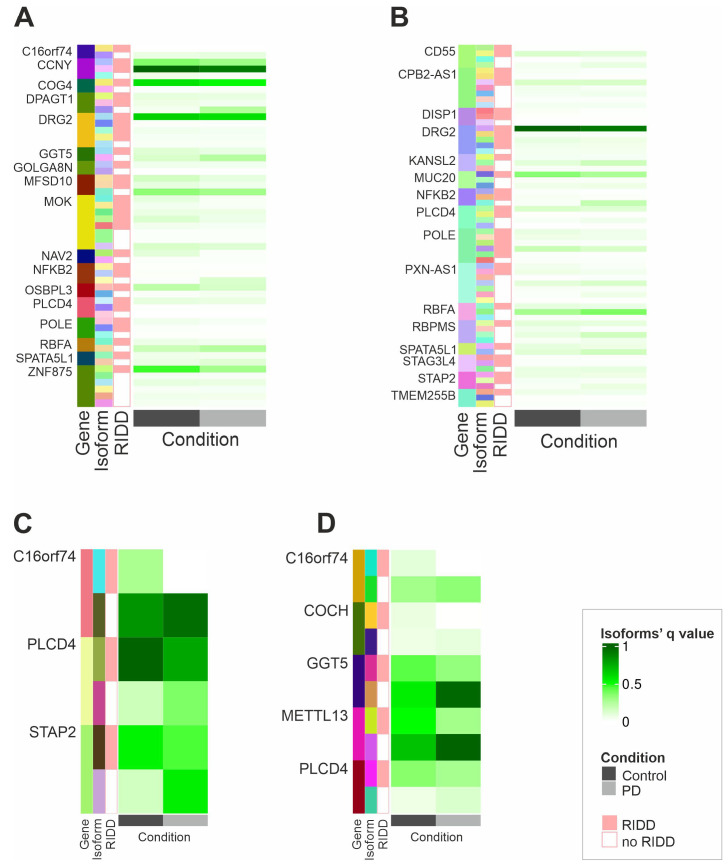
IRE1’s RIDD activity and differential isoform switching. (**A**–**D**) Genes whose isoforms display “A3 gain” events (**A**), “A5 gain” events (**B**), “domain loss” events (**C**), or “transcript non-coding” events (**D**); exhibit differential RIDD recognizability; and have at least one downregulated RIDD transcript are shown. Different isoforms for a single gene are represented in different colors.

**Figure 5:**
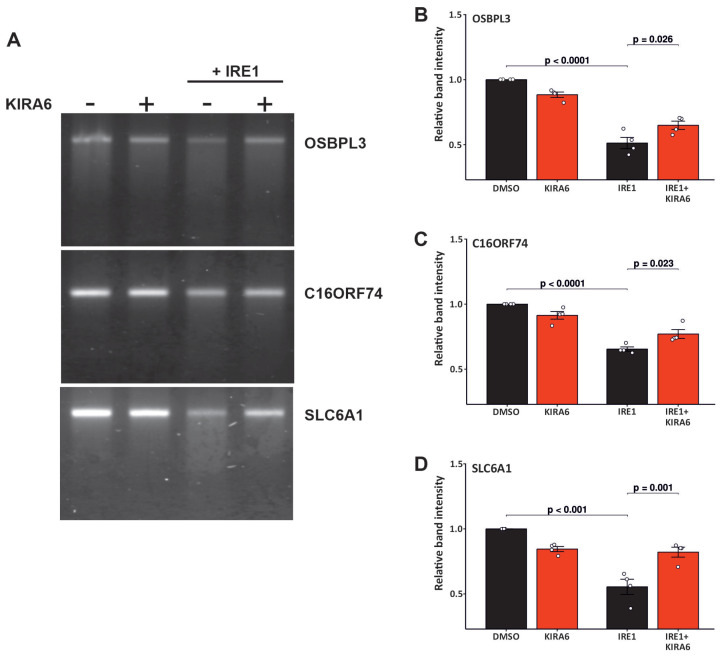
IRE1 cleaves *OSBPL3*, *SLC6A1*, and *C16orf74* mRNAs in vitro. (**A**) In vitro-transcribed *OSBPL3*, *C16orf74*, and *SLC6A1* mRNAs were incubated with 50 nM recombinant hIRE1α in the presence or absence of 50 μM of KIRA6. The gel images are representative of four independent experiments. The intensity of the bands for *OSBPL3* (**B**), *C16ORF74* (**C**), and *SLC6A1* mRNAs was quantified (**D**). The analysis was performed using an ANOVA followed by Tukey’s HSD test.

Building on our findings, we investigated how IRE1’s RIDD activity might influence the differential isoform usage between PD and control samples. Differential isoform usage refers to the different proportion of isoforms used by genes between different conditions [20,21]. This differential isoform usage can lead a specific splicing event to be enriched in one condition. We focused on transcripts with enriched isoform switching events, including ‘increased 5′ and 3′ UTR lengths’ (A3 and A5 gain), ‘domain loss’, and ‘transcript non-coding’. To determine whether these transcripts could be potential RIDD targets, we applied a previously published method (gRIDD) to identify RIDD targets, which are characterized by the presence of a CNGCNG sequence within a stem–loop structure [22]. Table S2 provides an overview of all of the candidate RIDD targets identified in our data, specifically from important features originating from the (a) isoform, (b) integrated, and (c) proteomics analyses. To refine this analysis, we retained only transcripts that were both potential targets of RIDD and significantly downregulated in patients with PD. We were particularly interested in genes whose isoforms showed differential recognizability of RIDD, that is, where one or more isoforms contained a potential RIDD target site while others did not, since splicing events such as increased 3′UTR (Figure 4A) and 5′UTR (Figure 4B) lengths, “domain loss” (Figure 4C), and “a transcript becoming non-coding” (Figure 4D) could influence the presence of a RIDD target site. We were interested to see whether this phenomenon, i.e., the presence of a potential RIDD target site, was associated with the presence of enriched splicing events. Therefore, we performed a correlation analysis for each gene that had both (a) the existence of an enriched splicing event and (b) differential RIDD recognizability to see the association of the potential RIDD target site with the splicing event observed. Pairs that passed the absolute threshold of 0.5 are shown in Figure S3. This analysis revealed that an increased UTR length significantly correlated with the presence of a potential RIDD target site for the majority of the genes displayed in Figure 4A,B, such as Component Of Oligomeric Golgi Complex 4 (*COG4*), CPB2 Antisense RNA 1 (*CPB2-AS1*), Golgin A8 Family Member N (*GOLGA8N*), and others. All significantly correlated features can be seen in Figure S3. Similarly, all genes with “domain loss” or “non-coding transcript” events also showed a strong correlation with potential RIDD target sites; see Figure S3 and Figure 4C,D. We then applied different metrics to analyzing the association of potential RIDD target sites with the existence of an enriched splicing event (Figures S4 and S5). Our data suggest an association between the occurrence of a potential RIDD target site and enriched splicing events, both at the level of individual genes and across the entire gene set.

### 2.4. IRE1 RNAse Activity Leads to the Degradation of *OSBPL3*, *C16orf74*, and *SLC6A1* mRNAs

To validate the effects observed downstream of IRE1 signaling, we conducted in vitro cleavage assays for three candidate transcripts: *OSBPL3*, *C16orf74* (Chromosome 16 Open Reading Frame 74), and *SLC6A1* (Solute Carrier Family 6 Member 1). As observed in Figure 2B, OSBPL3 was chosen based on its consistent downregulation across the transcriptomic, proteomic, integrative, and differential isoform usage analyses. C16orf74 was selected due to its involvement in three out of four enriched splicing events identified in our isoform analysis. SLC6A1 was included due to supporting evidence from both transcriptomic and proteomic datasets. As we can see in Figure 5, incubation of these mRNAs with IRE1 caused the degradation of all of these targets, and KIRA6 (Kinase-Inhibiting RNase-Attenuators 6), an IRE1 inhibitor, inhibited this degradation. We did not observe distinct bands that could indicate discrete cleavage products, suggesting that IRE1-mediated RIDDLE (a form of RIDD) may be responsible [22]. Quantification of the results shows statistically significant degradation of *OSBPL3*, *C16orf74*, and *SLC6A1*; Figure 5B–D.

### 2.5. The Differential Impact of RIDD Activity on Protein Abundance in PD

**Figure 6:**
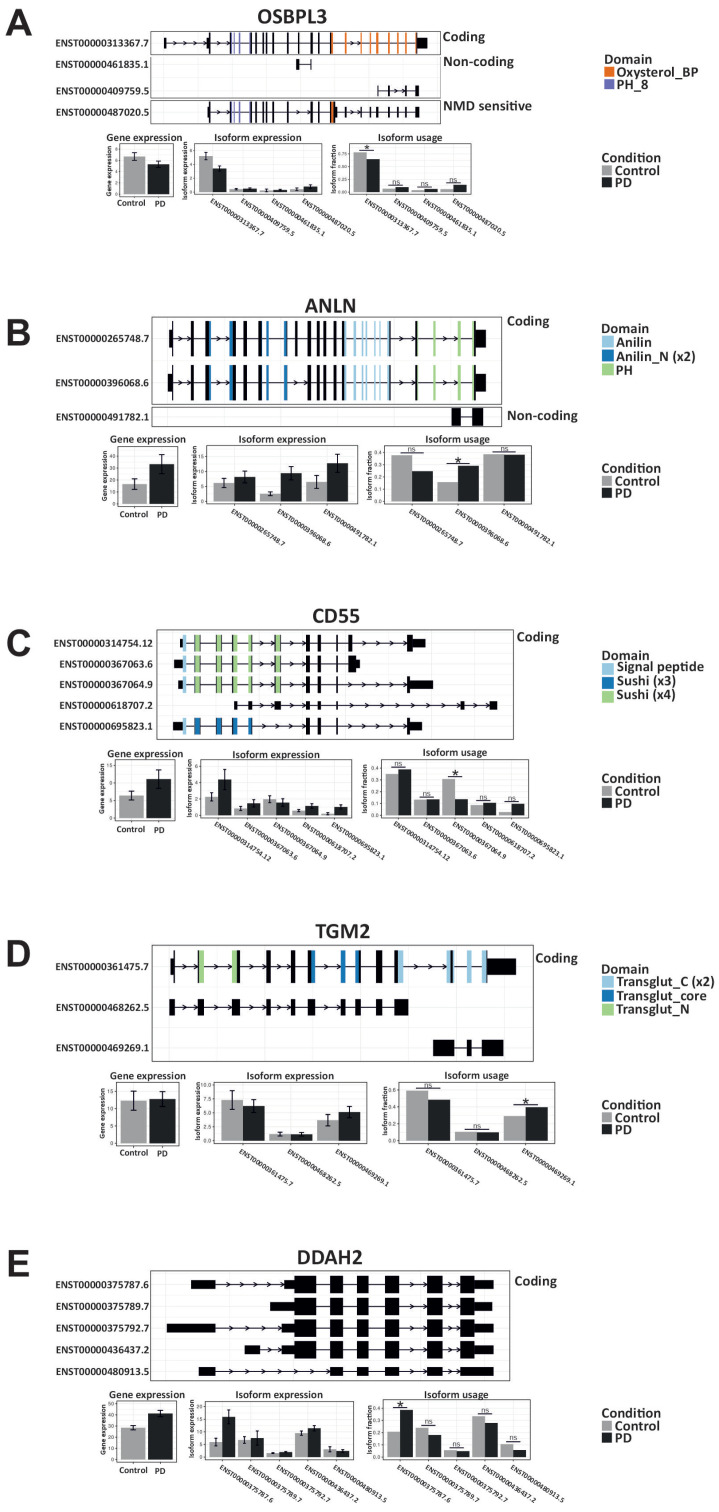
The differential impact of RIDD activity on protein abundance in PD. (**A**–**E**) RIDD features found in both proteomics and transcriptomics datasets for OSBPL3 (**A**), ANLN (**B**), CD55 (**C**), TGM2 (Transglutaminase 2) (**D**), and DDAH2 (**E**). Each panel shows the annotated isoforms of the gene, including coding potential and known domains (e.g., protein motifs, signal peptides). The lower sections report the gene-level and isoform-level log_2_ fold changes and differential isoform usage (dIF) between control and treatment conditions. Isoform expression represents the absolute abundance of a transcript, and isoform usage refers to its relative contribution to the total expression of its gene. The statistical analysis was performed using generalized linear models (GLMs), as implemented in IsoformSwitchAnalyzeR, with cutoffs of a false discovery rate (FDR) < 0.05 and |dIF| > 0.10. Statistically significant results are denoted with * and not significant with ns.

IRE1’s RNAse activity targets specific mRNAs, leading to their degradation. In both the transcriptomics and proteomics datasets, OSBPL3 is the only one whose total gene and protein expression is downregulated in PD, suggesting it may be downstream of IRE1’s RNAse activity (Figure 6A). Figure 6B–E highlight features with downregulated isoforms predicted to be targets of RIDD, while their overall gene or protein expression is upregulated in the PD samples. One possible explanation is that these genes may also be regulated by XBP1s to increase their expression. We analyzed the promoter sequences of these genes for potential XBP1s-binding sites and found that each gene had at least one (Table S3 and Figure 2C). This finding highlights an added layer of complexity in IRE1 signaling in PD, showing that *XBP1s* and RIDD can share common targets, whose expression is ultimately influenced by their interaction.

### 2.6. Activity of the XBP1 Axis in PD Samples

**Figure 7:**
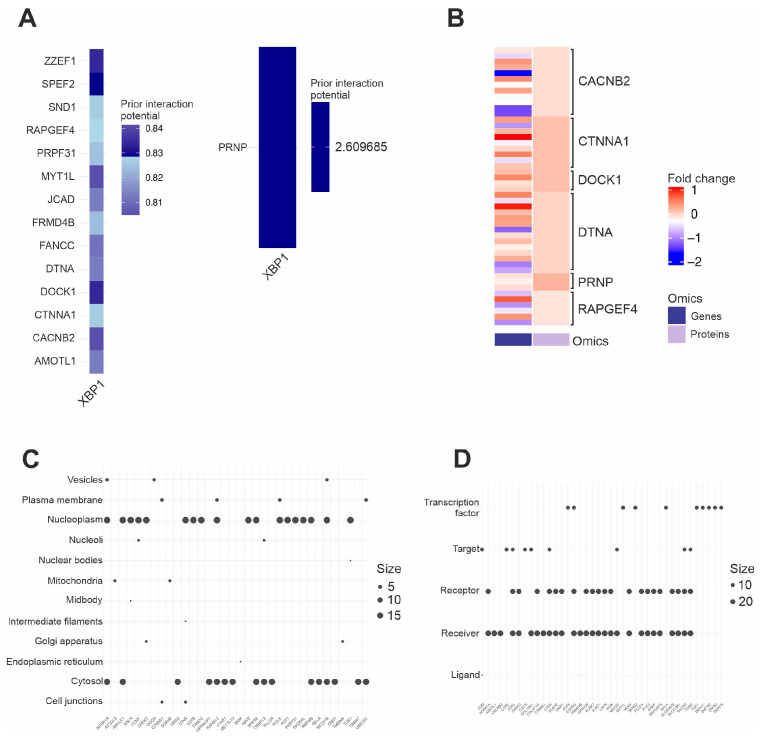
The XBP1s and RIDD axes in PD. (**A**) Targets of XBP1s present in our data, along with their prior interaction scores retrieved from MOI using omnipathR. *PRNP* is shown separately because of its relatively high interaction potential score. (**B**) Fold changes regarding transcriptomics and proteomics data for XBP1s targets. (**C**) The most enriched subcellular locations of targets of both the XBP1s and RIDD axes in the GO database. (**D**) The most enriched roles that these features have in the signaling pathways where they reside.

As demonstrated previously (Figure 3B), the observed isoform patterns of *XBP1* align with expected changes due to IRE1 activation. Building on this, we examined the known XBP1s targets present in our data and retrieved their interaction scores (for XBP1s interactions with the target promoters) using the omnipathR module in MultiOmicsIntegrator [12,23] (Figure 7A). We then assessed the fold changes in these targets in both the transcriptomics and proteomics datasets and kept only the common features that were significantly deregulated at the proteomic level (Figure S6). None of the downstream targets of XBP1s were found to be significantly deregulated by the isoform analysis, possibly due to the inadequacy of this analytical approach in the context of XBP1 signaling. As shown in Figure 7B, while some isoforms of these genes are downregulated, their proteins are upregulated, which is consistent with our expectations; for example, the CACNB2 (Calcium Voltage-Gated Channel Auxiliary Subunit Beta 2), CTNNA1 (Catenin Alpha 1), and DOCK1 (Dedicator Of Cytokinesis 1) proteins are upregulated although some of their transcripts are downregulated.

### 2.7. Subcellular Localization and Molecular Roles of IRE1 Downstream Targets

After confirming the activity of both the RIDD and XBP1s arms of IRE1, we investigated whether the identified targets exhibited preferences for specific subcellular locations or were associated with particular molecular categories within the signaling pathways in which they participated (e.g., transcription factors, ligands, receptors, etc.). The most enriched subcellular locations were the nucleoplasm and the cytosol, followed by the vesicles, the plasma membrane, the mitochondria, the Golgi apparatus, and the cell junctions (Figure 7C). These findings align with previous studies on IRE1 downstream targets [24]. In terms of their roles within the signaling pathways, Figure 5D depicts the most enriched molecular categories, which were receptors and interactors of receptors, (e.g., CD55 and CHEK2 (Checkpoint Kinase 2), respectively), followed by transcription factors and targets of transcription factors (e.g., E2F6 (E2F Transcription Factor 6) and CCN5 (Cellular Communication Network Factor 5), respectively).

### 2.8. The Pathway Enrichment Analysis of IRE1 Downstream Signaling Arms in PD

**Figure 8:**
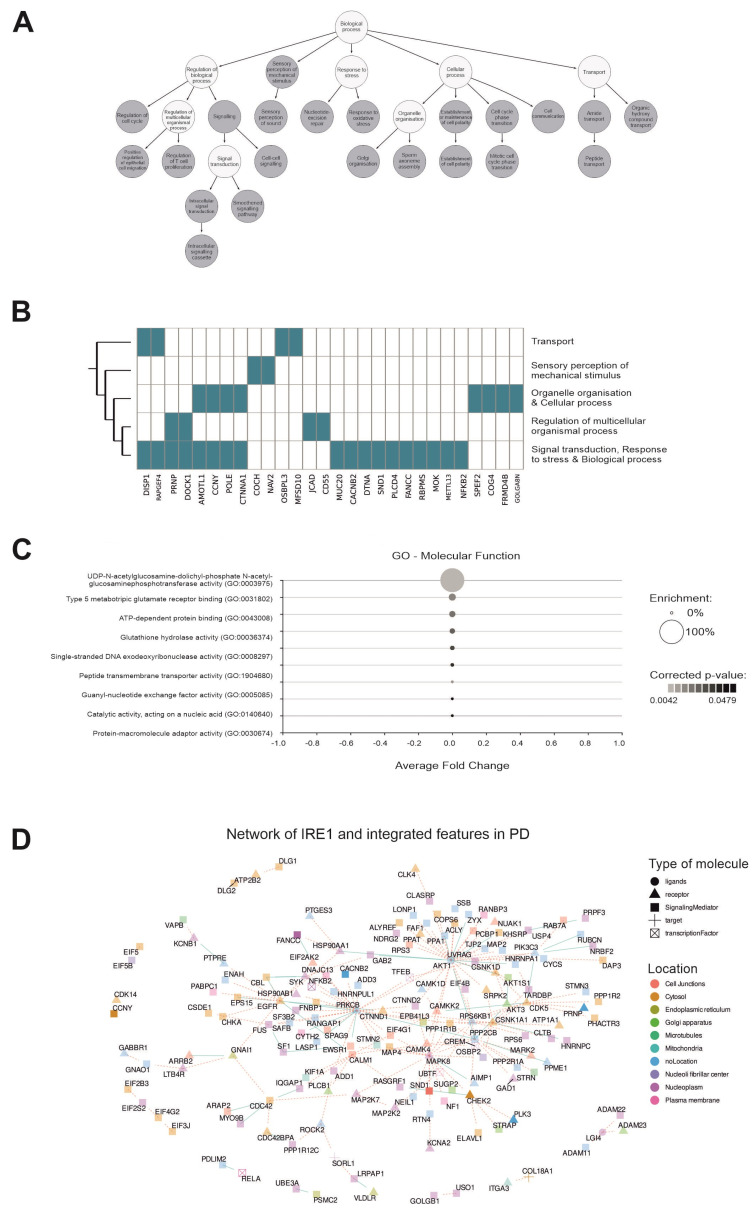
A pathway enrichment analysis was conducted using biotranslator from MOI on the XBP1s and RIDD signaling arms downstream of IRE1. (**A**) The most enriched biological processes in which the IRE1 downstream signaling arms participate are shown. (**B**) The distribution of hub genes across these processes. (**C**) The most enriched molecular functions. (**D**) The protein–protein interaction network of the IRE1 downstream signaling arms, along with the important features computed using the MCIA and information about the subcellular locations and roles in the signaling pathway (a high color intensity indicates a positive association with IRE1 signaling).

We conducted a pathway enrichment analysis including both arms of IRE1 identified in the data. This analysis revealed several enriched biological processes, including oxidative stress, signal transduction, Golgi organization, cell–cell communication, and T-cell proliferation (Figure 8A,B), all of which have previously been linked to PD [23,25,26,27]. Among the enriched molecular functions, we found glutathione hydrolase activity (Figure 8C), which catalyzes the hydrolysis and transpeptidation of many gamma-glutamyl compounds, including glutathione, a function previously associated with PD pathology [28]. Finally, we explored how the signaling arms downstream of IRE1 interacted with the important features uncovered through the MCIA by computing the protein–protein interaction network. Figure 8D depicts this network, highlighting the location of these features and their roles in signal transduction. This showcases the influence of IRE1 signaling on the most descriptive features obtained through the MCIA and highlights its importance in the pathology of PD.

## 3. Discussion

In this study, we conducted a meta-analysis of transcriptomic and proteomic data from a previously published study on PD [13]. Building on the original findings, we added a post-transcriptional layer of analysis by exploring isoform switching events. Several enriched isoform switching genes have known links to neurodegeneration (e.g., *ANLN*, *DDAH2*, *CD55*, and *DRG2*) [14,15,16,29,30,31], supporting the biological relevance of our approach. We examined IRE1’s involvement in these transcriptional changes further and found evidence suggesting its regulatory influence on isoform switching in PD. Our data indicate an association between potential RIDD target sites and enriched splicing events, both at the individual and global gene levels. While our correlation-based analyses (chi-squared, Cramér’s V, mutual information) suggest a link between alternative splicing and RIDD susceptibility, these findings may be influenced by factors such as the transcript abundance, isoform dominance, RNA stability, and context-specific expression. Whether isoform switching creates RIDD sites or is a result of IRE1 activity remains an open question. However, it should be noted that other studies have previously linked splicing events with altered post-transcriptional-related processes [32] and especially IRE1-RIDD activity [33].

One of the most promising findings of our analysis was the confirmation of RIDD activity for three genes, namely *OSBPL3*, *C16orf74*, and *SLC6A1*, none of which have previously been linked to PD. *OSBPL3* encodes a member of the oxysterol-binding protein (OSBP) family, which are lipid transport proteins. It has been reported to play a significant role in several cancers that may be linked to its ability to regulate the immune environment. It has been implicated in promoting ER stress and stemness in triple-negative breast cancer, contributing to tumor metastasis and resistance to chemotherapy, and additionally, it has been involved in immune response [34,35]. *C16orf74* encodes a microprotein with an unknown function that is implicated in cancer pathogenesis and prognosis. It has recently been reported to promote thermogenesis in brown adipose tissue [36]. It has also been associated with the modulation of immune responses in the tumor microenvironment, where its expression may inversely correlate with immune cell infiltration, potentially diminishing anti-tumor immunity in head and neck carcinoma [37]. *SLC6A1* encodes the gamma-aminobutyric acid (GABA) transporter 1 (GAT-1), which is critical for the reuptake of GABA, the principal inhibitory neurotransmitter in the central nervous system [38]. Thus, it plays a key role in normal neurological function, and genetic alterations that impact GABA clearance from the synaptic cleft can lead to various neurodevelopmental disorders and epileptic conditions [38]. Notably, deep brain stimulation, an effective therapy for PD, was recently shown to alleviate PD symptoms through the modulation of GABA release [39]. The identification of these genes as potential RIDD targets presents an exciting opportunity to investigate their roles in PD progression and assess their potential as therapeutic targets in PD.

Similarly, activation of the IRE1/XBP1s axis is a critical component of the cellular response to ER stress. There is some evidence that activation of IRE1-XBP1s signaling may be neuroprotective in models of PD [40,41]. Its activation promotes neuroprotection through mechanisms such as enhanced autophagy and regulation of protein homeostasis [41]. However, the context and duration of its activation are crucial, as deregulation can lead to neuronal death. In our data, we examined the expression of known XBP1s targets and confirmed that the IRE1/XBP1s axis was active in the PD samples. Of particular interest were some RIDD candidates that were also identified in the proteomics dataset and were upregulated in both omics layers but not necessarily at the isoform level. We found at least one predicted XBP1s-binding site in the promoters of these genes, suggesting that XBP1s and RIDD may co-regulate shared targets, adding complexity to IRE1 signaling in PD. Another plausible explanation is that the differential susceptibility of isoforms to RIDD enables IRE1 to selectively target certain transcripts, thereby increasing the accessibility of ribosomes to the remaining isoforms and potentially resulting in the enhanced translation of transcripts lacking a RIDD target site. Interestingly, the pathway enrichment for both arms downstream of IRE1 revealed processes such as immune response and metabolism that are well known in PD pathology [42], further validating our findings.

One limitation of our study is the exclusive focus on the prefrontal cortex, which, of course, is not the primary brain region affected in PD. Investigation of the substantia nigra pars compacta region would be more relevant for future studies. Furthermore, the application of cutting-edge technologies such as single-cell and spatial transcriptomics could provide a more complete understanding of IRE1 signaling across brain regions, as well as between the neuronal and glial cells.

Although our study provides a comprehensive computational analysis of PD samples, experimental validation is essential to confirm the functional relevance of our findings. In particular, follow-up studies should investigate the biological consequences of modulating IRE1 activity or perturbing downstream effectors (such as OSBPL3) in PD-relevant cellular and in vivo models. Such work will be crucial to move from computational inference to a mechanistic understanding.

It is also important to recognize that IRE1 does not act in isolation but cooperates with other ER stress sensors, PERK and ATF6, to orchestrate the unfolded protein response [43]. For example, ATF6 promotes XBP1 expression, thus influencing IRE1-XBP1s signaling [11]. This interplay among UPR branches in PD is complex and warrants further investigation. Our study provides a foundation for such work by focusing on IRE1, and future studies could help uncover how coordinated ER stress signaling contributes to neurodegeneration.

Here, we present evidence for the relevance of IRE1 signaling in PD pathogenesis, highlighting activity in both its XBP1s and RIDD arms. These pathways influence processes like immune response and oxidative stress. We identified novel RIDD targets, some of which may also be regulated by XBP1s, suggesting an intricate balance between the two arms. Our findings lay a strong foundation for future research into IRE1-driven mechanisms in PD.

## 4. Materials and Methods

### 4.1. The Transcriptomics and Proteomics Data

Our analysis was based on the previously published study GSE68719 [13]. We selected samples that included both proteomics and transcriptomics data for an individual control or PD patient sample to a total of 24 samples. All samples were from males. The age at the time of death was 79.50 years (61–97) for the controls and 76.83 years (64–88) for the PD patients. The samples were from frozen brain tissue from the prefrontal cortex, Brodmann Area 9. The details on the selected samples are provided in Table S1.

### 4.2. The Transcriptomics Analyses

All transcriptomics analyses were conducted using our Nextflow-based pipeline, MultiOmicsIntegrator [12]. The data were retrieved using their corresponding SRA codes. Adapter sequences were trimmed, and quality control was performed using FastQC [44]. Reads were aligned with the human reference genome (hg38) from Ensembl and assembled into transcripts using the splice-aware tool salmon [45].

The isoform switching analysis was conducted using IsoformSwitchAnalyzer [21], integrated within MultiOmicsIntegrator. This method groups isoforms based on their expression patterns across conditions and applies statistical tests to identify significant isoform switching events. Batch effects were accounted for in the linear model used during this analysis.

### 4.3. The Proteomics Analysis

The proteomics data were analyzed independently and in conjunction with transcriptomics data using MOI. The data underwent filtering, normalization, and batch effect correction. The resulting clean data matrix was utilized for both the differential expression and pathway enrichment analyses, as well as for its integration with the MCIA [46].

### 4.4. Integration of Omics

MultiOmicsIntegrator incorporates data-driven integration methods, such as MCIA, which was used for the integration of omics. This approach first centers and scales the data and then identifies shared patterns through a joint covariance analysis. Optimization is applied to calculating the coefficients for each feature, quantifying their contribution to the shared structures. The method constructs latent variables (or components) that represent these shared patterns, with optional dimensionality reduction if necessary. Pathway enrichment analysis was performed with biotranslator from MOI (see Tables S4 and S5).

### 4.5. IRE1 Scoring

To score the control and PD patient samples based on IRE1 activity, all available transcript expression data were filtered and normalized using transcripts per kilobase million (TPM). Then, previously published RIDD targets (see Table S6) and isoforms of XBP1 were distributed into quantiles based on their expression in our data. RIDD activity leads to transcript degradation, so a lower expression means a higher quantile. Then, for each sample, an IRE1 activity score was calculated using the sum of quantiles divided by the number of features.

### 4.6. gRIDD

To identify new RIDD targets, we employed the gRIDD algorithm [22] integrated within MultiOmicsIntegrator. This algorithm searches for the motif NNCNGCNGNN within transcript sequences. If the motif is detected, the window is extended, and the probability of the sequence forming a stem–loop structure is calculated, i.e., the probability of it being a RIDD target site.

### 4.7. Comparison of the Isoforms

A comparison of the sequences of the different isoforms was performed using the online tool the UVA Fasta server with the default parameters.

### 4.8. XBP1 Targets

XBP1s targets were identified using FIMO [47], and promoter binding sequences were obtained from the Jaspar database [48].

### 4.9. The In Vitro mRNA Cleavage Assay

pcDNA3.1[+] plasmids expressing full-length *OSBPL3* (ENST00000313367), *C16orf74* (ENST00000284245), and *SLC6A1* (ENST00000645598) were purchased from Genscript, Piscataway, NJ, USA. The plasmids were linearized through digestion with the appropriate restriction enzymes (BsaBI for OSBPL3 and NotI-HF for C16orf74 and SLC6A1), followed by the removal of salts and enzymes using a column-based Nucleospin PCR cleanup system (Macherey-Nagel, Düren, Germany, 740609.50) according to the manufacturer’s protocol. The linearized plasmids served as templates for in vitro transcription with the T7 RiboMAX Express Large Scale RNA Production System (P1320, Promega, Madison, WI, USA) following the manufacturer’s instructions. The RNA was purified with the RNeasy mini kit (74106, QIAGEN, Hilden, Germany). Recombinant IRE1 protein (11905-HNCB, Sino Biological, Beijing, China) was pre-incubated in RNAse buffer solution (20 mM HEPES, pH = 7.5; 1 mM MgOAc2; and 50 mM KOAc; 2 mM DTT) in the presence of DMSO (D2650, Sigma-Aldrich, Burlington, MA, USA) or KIRA6 (5.32281, Sigma-Aldrich, Burlington, MA, USA) for 15 min at room temperature. Then, 500 ng of RNA was added to the mix to a final volume of 10 mL. The mix was incubated for 30 min at 37 °C, followed by 8 min at 65 °C to stop the IRE1 activity. Cleaved RNAs were separated in a 1.5% low-melt agarose gel (16520-050, Invitrogen, Carlsbad, CA, USA) in 1X MOPS buffer (4.185 g of MOPS, 0.41 g of NaOAc, and 0.372 g EDTA in 1 L of H_2_O; pH = 7) for 90 min at 50 V. Images were taken using a D-Digit gel scanner (Li-cor Biosciences, Lincoln, NE, USA).

## Data Availability

Data is contained within the article and Supplementary Material.

## Supplementary Materials

The following supporting information can be downloaded at https://www.mdpi.com/article/10.3390/ijms26146711/s1.

## Acronyms

6-OHDA	6-hydroxydopamine
ANLN	Anillin, Actin Binding Protein
ATSS	Alternative Transcript Start Site
ATTS	Alternative Transcript Termination Site
BiP	Binding Immunoglobulin Protein
C16orf74	Chromosome 16 Open Reading Frame 74
CACNB2	Calcium Voltage-Gated Channel Auxiliary Subunit Beta 2
CCN5	Cellular Communication Network Factor 5
CD55	CD55 Molecule (Cromer Blood Group)
CHEK2	Checkpoint Kinase 2
COG4	Component Of Oligomeric Golgi Complex 4
CPB2-AS1	CPB2 Antisense RNA 1
CTNNA1	Catenin Alpha 1
DA	Dopaminergic neurons
DDAH2	DDAH Family Member 2, ADMA-Independent
DOCK1	Dedicator Of Cytokinesis 1
DRG2	Developmentally Regulated GTP Binding Protein 2
E2F6	E2F Transcription Factor 6
ER	Endoplasmic Reticulum
ES	Exon Skipping
GABA	Gamma-Aminobutyric Acid
GAT-1	Gamma-Aminobutyric Acid Transporter 1
GO	Gene Ontology
GOLGA8N	Golgin A8 Family Member N
IR	Intron Retention
IRE1	Inositol Requiring Enzyme 1
KIRA6	Kinase-Inhibiting RNase-Attenuator 6
MCIA	Multiple Co-Inertia Analysis
MEI	Multiple Exon Inclusion
MES	Multiple Exon Skipping
METTL13	Methyltransferase 13, EEF1A N-Terminus And K55
MOI	MultiOmicsIntegrator
MPP+	1-methyl-4-phenylpyridinium
mRNA	Messenger Ribonucleic Acid
OSBPL3	Oxysterol Binding Protein Like 3
PD	Parkinson’s Disease
PERK	Protein Kinase (RNA)-like Endoplasmic Reticulum Kinase
PLCD4	Phospholipase C Delta 4
PRNP	Prion Protein (Kanno Blood Group)
RIDD	Regulated IRE1-Dependent Decay
RNase	Endoribonuclease
SLC6A1	Solute Carrier Family 6 Member 1
STAP2	Signal Transducing Adaptor Family Member 2
TGM2	Transglutaminase 2
TJP2	Tight Junction Protein 2
TPM	Transcripts Per Kilobase Million
TH	Tyrosine Hydroxylase
UPR	Unfolded Protein Response
UTR	Untranslated Regions
XBP1	X-box Binding mRNA 1
XBP1s	Spliced XBP1
XBP1u	Unspliced XBP1
