# Supplementary material for: Multiomics Integration of Parkinson’s Disease Datasets Reveals Unexpected Roles of IRE1 in Its Pathology
Source: Int J Mol Sci. 2025 Jul 12;26(14):6711. doi: 10.3390/ijms26146711 (PMC12295087; doi:10.3390/ijms26146711)
Supplement: Supplementary file 1 [file ijms-26-06711-s001.zip › SupplementaryFigures_Pasat_et_al-PD.pdf]

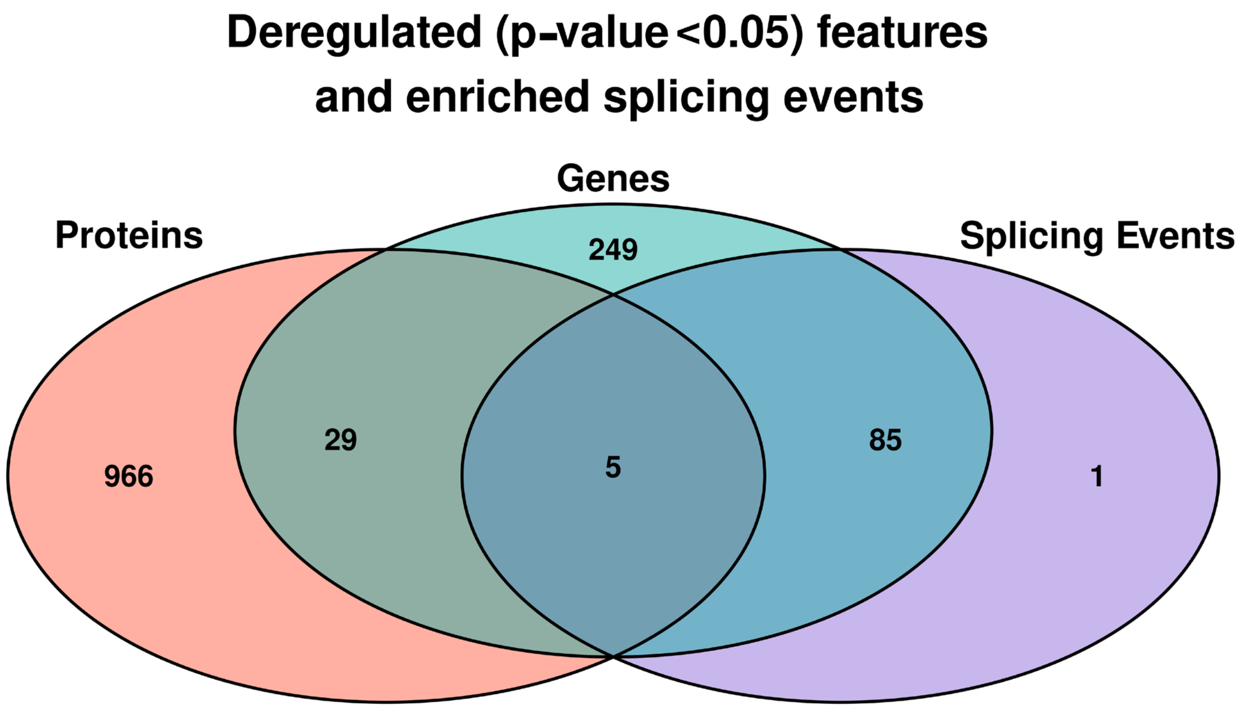

Figure S1: Venn Diagram of significantly differentially expressed features. Deregulated (p-value <0.05) genes, proteins, and their overlap with the enriched splicing events (i.e., A3 gain, A5 gain, Transcript non-coding, Domain loss.

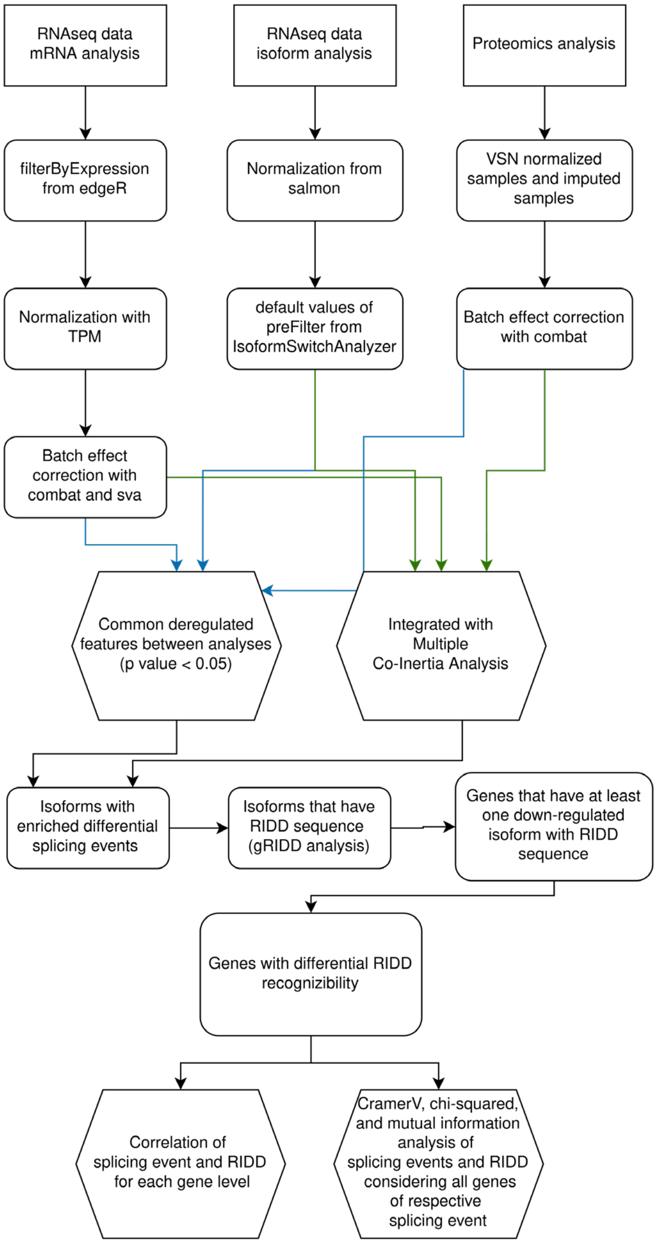

Figure S2: Analytical steps presented in the manuscript. Steps up to and including gRIDD analysis were executed with MOI. Subsequent steps were performed in R.

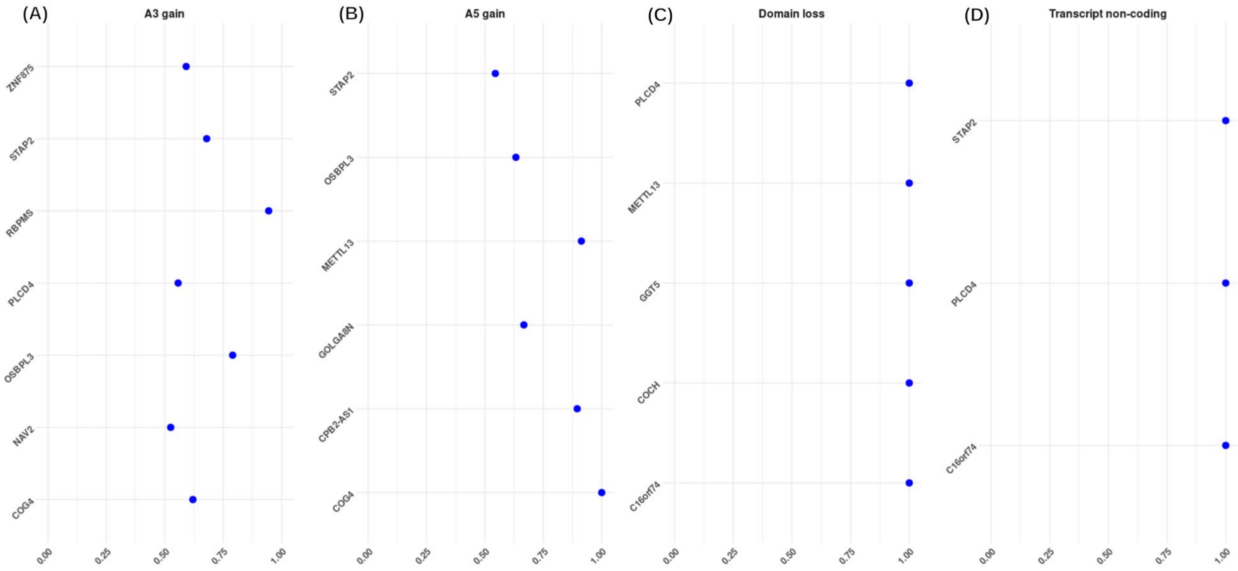

Figure S3: Correlation scores of the presence of a RIDD motif and occurrence of a splicing event for each gene. Genes that display i) enriched splicing events, ii) differential RIDD recognizability (i.e., some isoforms of a gene display RIDD while some isoforms do not) and iii) at least one isoform that displays downregulation of the RIDD motif are shown. The threshold was set at correlation >|0.5|.

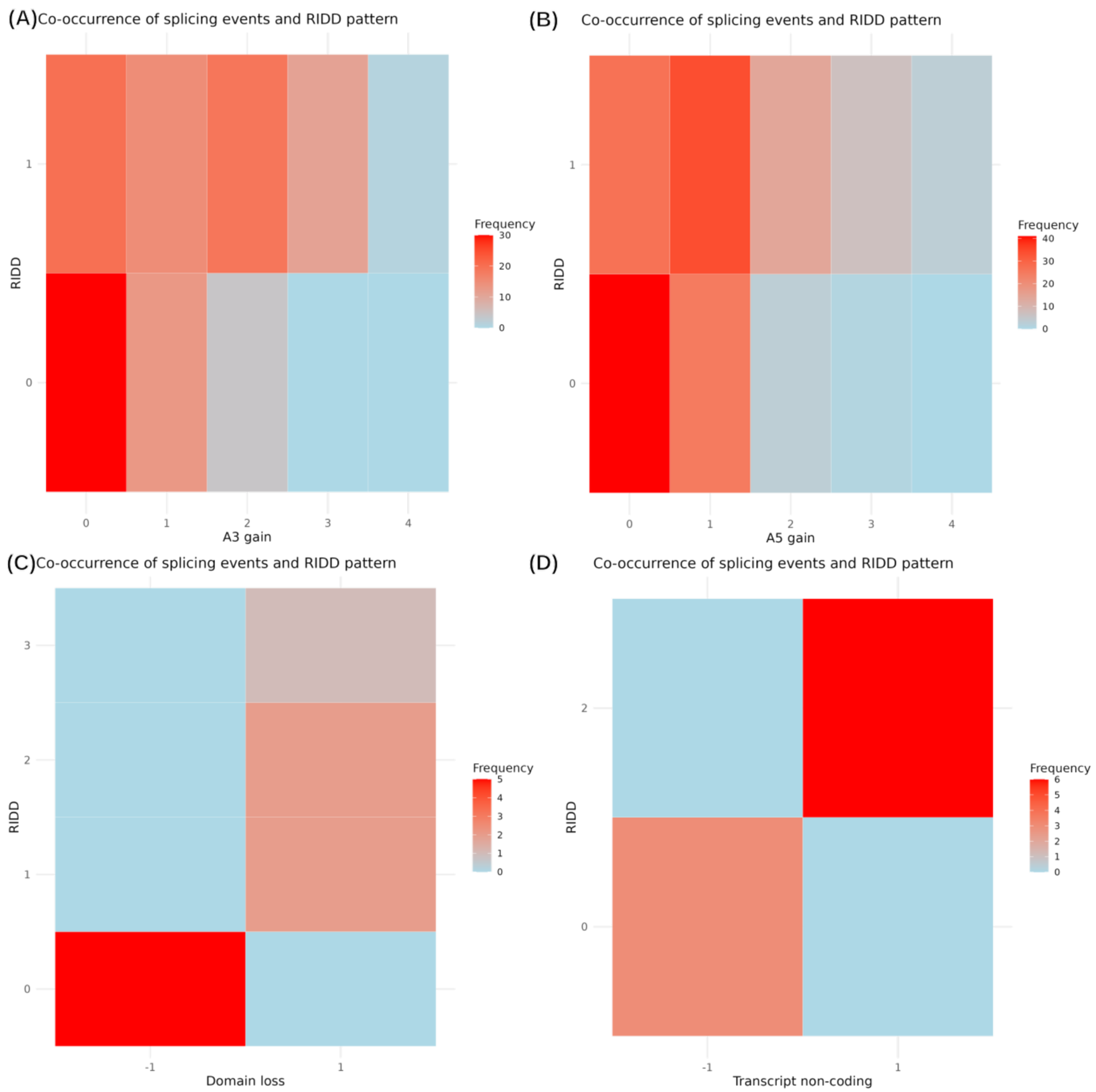

Figure S4: Contingency matrices of genes that show correlation of RIDD motif enriched splicing event. (A-D) Contingency matrices of frequency of RIDD patterns (Y axis) and enriched splicing events (X axis). Color coding indicates the frequency of isoforms within each category, reflecting the combination of both attributes. In panels A and B, the X-axis shows the extent of A3 and A5 gain, while the Y-axis indicates the number of identified RIDD sequences. In panels C and D, a score of +1 denotes isoforms with both domain loss and downregulation (consistent with RIDD activity), while -1 indicates domain loss coupled with upregulation.

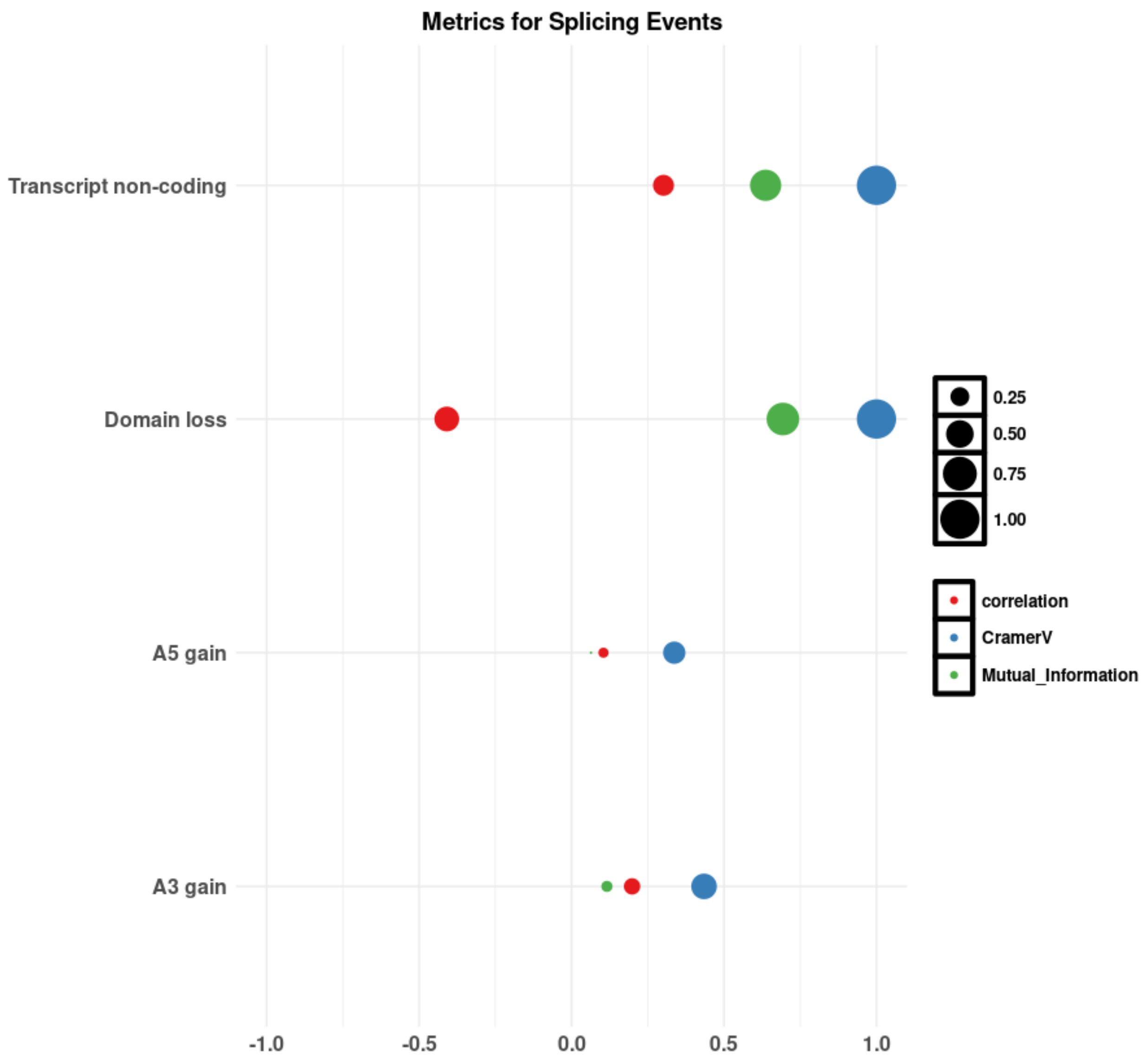

Figure S5: Association metrics of RIDD motif and enriched splicing events. Different association metrics of RIDD motif presence and splicing events occurrences for each splicing event based on the whole set of isoforms. Circle size reflects the strength of the association, while colors denote the metric used—correlation estimates derived from Chi-squared analysis, Cramér's V, or mutual information—each calculated across the full feature set.

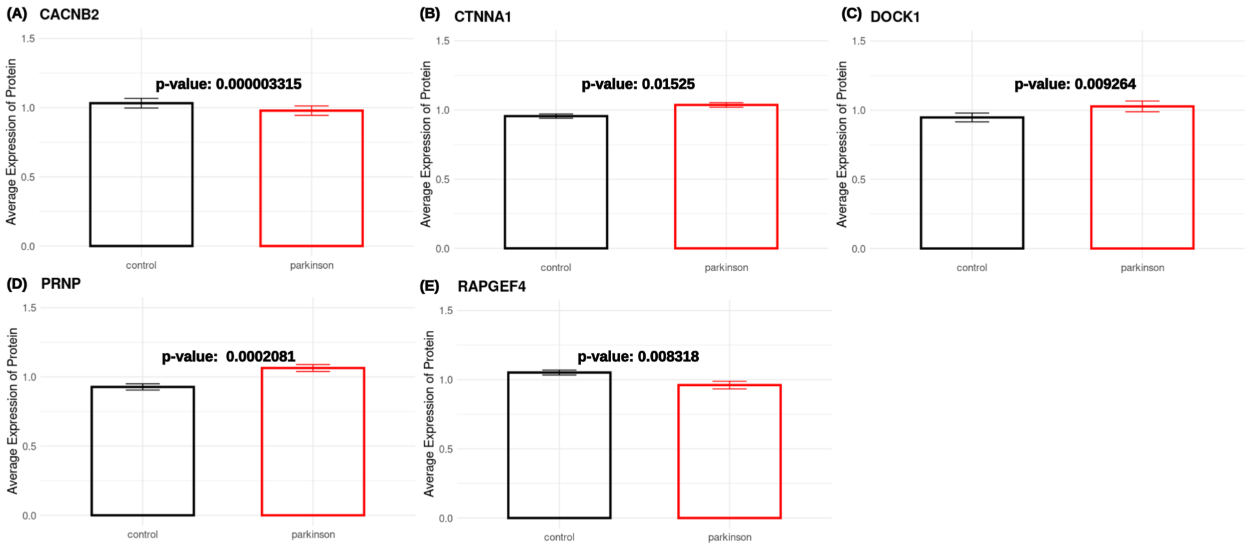

Figure S6: (A-E) Bar-plots of significantly differentially expressed (p-value < 0.05) proteins downstream of XBP1 signaling. Differential expression was performed with RankProd.
